# Diagnostic Superiority of Transperineal Combined Fusion Biopsy Versus Transrectal Ultrasound-Guided Biopsy: Lower Upgrading Rates and Better Concordance with Post-Surgical Histopathology

**DOI:** 10.3390/jcm14165698

**Published:** 2025-08-12

**Authors:** Emil Kania, Maciej Janica, Bartłomiej Kazimierski, Michał Wiński, Paweł Samocik, Robert Kozłowski, Wojciech Modzelewski, Mateusz Cybulski, Jacek Robert Janica

**Affiliations:** 1 Department of Urology, Śniadeckiego Voivodeship Hospital in Bialystok, 26 Sklodowskiej-Curie St., 15-278 Bialystok, Poland; b.kazimierskii@gmail.com (B.K.); mw.winski@gmail.com (M.W.); pawelsamocik@gmail.com (P.S.); rkozlowski12@gmail.com (R.K.); 2 Department of Radiology, Śniadeckiego Voivodeship Hospital in Bialystok, 26 Sklodowskiej-Curie St., 15-278 Bialystok, Poland; maciejjanica@wp.pl; 3 Department of Paediatric Radiology, Medical University of Bialystok, 17 Waszyngtona St., 15-274 Bialystok, Poland; wojciech.modzelewski@umb.edu.pl (W.M.); jrjanica@gmail.com (J.R.J.); 4 Department of Integrated Medical Care, Medical University of Bialystok, 7A Sklodowskiej-Curie St., 15-096 Bialystok, Poland; mateusz.cybulski@umb.edu.pl

**Keywords:** prostate biopsy, fusion biopsy, targeted biopsy, systematic biopsy, histopathological concordance

## Abstract

**Background/Objectives:** Accurate histopathological grading of prostate cancer at the time of biopsy is essential for guiding treatment decisions and minimizing the risks of both overtreatment and undertreatment. A key challenge in prostate cancer diagnostics is the phenomenon of upgrading, wherein the cancer appears more aggressive in the radical prostatectomy specimen than initially indicated by biopsy. Such discrepancies can compromise therapeutic planning. This study investigates whether transperineal combined fusion biopsy (ComBx), incorporating MRI-targeted and systematic sampling, achieves greater concordance with final prostatectomy histopathology compared to conventional transrectal ultrasound-guided systematic biopsy (TRUS-Bx). **Methods:** This retrospective cohort study analyzed 500 men aged 46 to 79 years (mean age 65) who underwent prostate biopsies between 2017 and 2022 at a single tertiary institution. Patients were stratified into two groups: 250 underwent TRUS-Bx using a 12-core systematic approach, and 250 underwent ComBx guided by software-based MRI–ultrasound fusion targeting PI-RADS ≥ 3 lesions, followed by systematic sampling. Histopathological grading from biopsies was compared with final pathology following radical prostatectomy. Concordance, upgrading, and downgrading rates were analyzed using appropriate statistical methods. **Results:** Prostate cancer was diagnosed in 113 patients in the TRUS-Bx group and 152 in the ComBx group. Among these, 89 TRUS-Bx and 68 ComBx patients underwent radical prostatectomy at our center. Histological upgrading occurred statistically significantly more often in the TRUS-Bx group (35%) compared to the ComBx group (16%) (*p* = 0.004). Concordance between biopsy and prostatectomy grading was statistically significantly higher in the ComBx group (63%) than in the TRUS-Bx group (49%) (*p* = 0.042). No significant difference in downgrading rates was observed between groups. **Conclusions:** Transperineal combined fusion biopsy substantially improves concordance with final prostatectomy histology and significantly reduces the risk of upgrading compared to transrectal systematic biopsy. These findings support the adoption of ComBx as a more reliable diagnostic strategy for accurate grading of clinically significant prostate cancer, with implications for improving treatment precision and patient outcomes.

## 1. Introduction

Prostate cancer remains one of the most frequently diagnosed malignancies globally, accounting for approximately 1.47 million new cases and nearly 398,000 deaths worldwide in 2022 [1]. Early and precise diagnosis plays a critical role in ensuring optimal clinical outcomes by enabling tailored therapeutic strategies aimed at reducing morbidity associated with both disease progression and overtreatment. Histopathological examination following prostate biopsy is the definitive method for establishing a prostate cancer diagnosis, with disease severity classified using the Gleason scoring system and subsequently grouped according to the International Society of Urological Pathology (ISUP) grading system [2].

Accurate grading at the biopsy stage is pivotal, as it directly influences treatment decisions such as active surveillance, radical prostatectomy, radiotherapy, or systemic therapies. However, substantial discrepancies between biopsy-based ISUP grading and final pathology from radical prostatectomy specimens have been consistently reported, posing significant clinical challenges [3]. Such discrepancies, manifesting as either upgrading or downgrading of the disease, can lead to inappropriate clinical decisions. Upgrading, defined as a higher ISUP grade found at radical prostatectomy compared to initial biopsy, carries particular clinical importance because it indicates a greater disease aggressiveness than initially suspected, potentially resulting in undertreatment. Conversely, downgrading could lead to unnecessary aggressive interventions, exposing patients to avoidable risks and complications, notably urinary incontinence and erectile dysfunction.

Historically, prostate biopsy has predominantly involved transrectal ultrasound-guided biopsy (TRUS-Bx), employing systematic sampling. While widely accessible and cost-effective, TRUS-Bx exhibits limitations, including significant rates of infection, patient discomfort, and notable sampling errors due to random rather than lesion-targeted sampling. Consequently, this conventional method frequently results in histopathological discrepancies and suboptimal diagnostic accuracy.

Recently, multiparametric magnetic resonance imaging (mpMRI) has significantly advanced prostate cancer diagnosis by providing detailed anatomical and functional information that enables precise localization and characterization of suspicious lesions using the PI-RADS v2.1 framework [4]. MRI-guided targeted biopsies, performed through cognitive or software-based fusion or in-bore techniques, leverage mpMRI data to achieve higher precision in sampling clinically significant lesions [5]. Among these, transperineal combined fusion biopsy (ComBx), which integrates systematic sampling with MRI–ultrasound fusion-guided targeting, has emerged as a particularly promising and practical approach. It offers a strong balance between diagnostic accuracy, time efficiency, and cost, making it one of the most accessible modalities for routine clinical use while reducing sampling error [6].

Previous studies have supported the superior diagnostic performance of MRI-targeted biopsies compared to systematic biopsy alone; however, there remains variability in concordance rates reported across different studies and techniques [7,8,9]. Furthermore, comparative data specifically evaluating concordance between ComBx and TRUS-Bx with final radical prostatectomy pathology remain limited and sometimes conflicting, underscoring the need for additional studies to clarify these diagnostic relationships.

Therefore, the aim of this study was to rigorously assess and compare the histopathological concordance rates between biopsy-derived ISUP grading and radical prostatectomy specimens obtained from patients undergoing either TRUS-Bx or ComBx. Our primary objective was to evaluate whether ComBx provides superior diagnostic accuracy in clinical practice, thereby reducing the risks associated with incorrect prostate cancer grading. We hypothesized that ComBx would significantly improve concordance with radical prostatectomy histopathology, lower upgrading rates, and thus optimize therapeutic decision-making processes.

## 2. Materials and Methods

This single-center cohort study was conducted retrospectively at the Urology Department of Śniadecki Voivodeship Hospital in Bialystok from January 2017 to December 2022.

A total of 500 consecutive men aged between 46 and 79 years (mean age 65) who underwent prostate biopsy due to elevated prostate-specific antigen (PSA) levels, abnormal digital rectal examination (DRE), or suspicious findings on mpMRI were enrolled. Patients were divided into two groups, each consisting of 250 individuals, based on the biopsy method employed: the transrectal ultrasound-guided systematic biopsy (TRUS-Bx) group and the transperineal combined fusion biopsy (ComBx) group. Patients with an initial PSA level above 50 or those diagnosed with disseminated disease were excluded from the analysis.

All procedures were conducted under local anesthesia, using a periprostatic injection of 2% lidocaine. Tissue sampling was performed with an 18-gauge core needle mounted on an automatic biopsy gun. All biopsy specimens were processed according to standardized histopathological protocols. Clinically significant prostate cancer (csPCa) was defined as ISUP Grade ≥ 2 (Gleason score ≥3 + 4 = 7).

In the TRUS-Bx group, a standard 12-core systematic sampling protocol was used, with six cores taken from each lobe of the prostate (base, mid-gland, and apex) to ensure comprehensive coverage of all glandular zones.

In the ComBx group, prostate biopsy was performed using a combined approach incorporating both MRI-targeted biopsies (TBx) and systematic biopsies (SBx). Prior to biopsy, all patients underwent multiparametric MRI (mpMRI), including T2-weighted imaging (T2WI), diffusion-weighted imaging (DWI), apparent diffusion coefficient (ADC) mapping, and dynamic contrast-enhanced (DCE) imaging. Radiologic interpretation and PI-RADS categorization were conducted by radiologists from various diagnostic centers, with imaging acquired on MRI scanners from different manufacturers. Importantly, all scans selected for fusion biopsy were assessed in accordance with the standardized PI-RADS (Prostate Imaging Reporting and Data System) guidelines. A PI-RADS score was assigned to each lesion, and a corresponding suspicious lesion map was included with every imaging report to guide targeted sampling. Only lesions scored as PI-RADS ≥ 3 were considered for targeting. In cases of equivocal interpretation, findings were reviewed during interdisciplinary radiologic–clinical consultations involving both radiologists and urologists prior to biopsy. All MRI scans met the technical specifications outlined in the PI-RADS protocol. Targeted biopsies were performed using the Koelis^®^ Trinity software-based fusion platform (Version 4.8.2 (R1)), which integrates pre-acquired MRI data into real-time transrectal ultrasound to enable precise lesion localization. For each MRI-identified lesion, two to four targeted cores were obtained based on lesion size and anatomical location. This was followed by systematic sampling, during which 8 to 12 additional cores were evenly distributed across the peripheral and transition zones of both prostate lobes to minimize sampling error and enhance diagnostic accuracy. Patients with biopsy-confirmed prostate cancer subsequently underwent radical prostatectomy (RP) at our institution. The surgical approach—either open retropubic or robotic-assisted laparoscopic (da Vinci Surgical System^®^, Intuitive Surgical)—was selected according to patient and surgeon preference. Histopathological evaluation of all biopsy specimens and postoperative samples was performed by the same experienced pathologist, ensuring interpretative consistency and reducing intraobserver variability.

**Figure 1:**
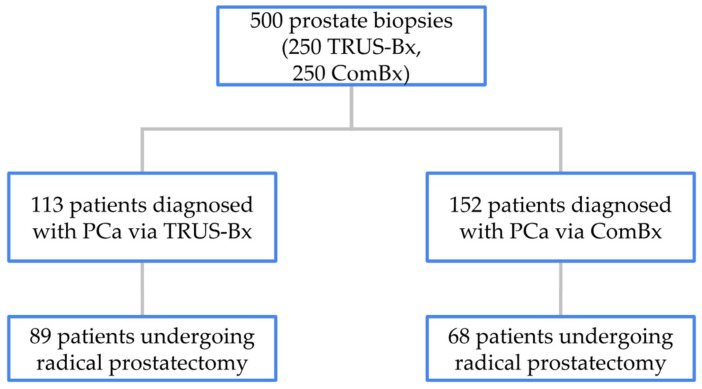
Distribution of patients treated for PCa based on biopsy method.

In the TRUS-Bx group, 113 patients were diagnosed with PCa, of whom 89 underwent RP at our institution. The remaining 24 patients received treatment at other medical centers or after the study’s data collection period. In the ComBx group, PCa was diagnosed in 152 patients, with 68 undergoing RP at our center and the remaining 84 treated either elsewhere or outside the timeframe of the study. The distribution of patients diagnosed and treated based on biopsy method is illustrated in Figure 1.

Histopathological concordance was evaluated by comparing the ISUP grade from biopsy specimens with the corresponding grade from radical prostatectomy specimens. Upgrading was defined as an increase in ISUP grade from biopsy to prostatectomy, indicating a more aggressive tumor than initially assessed. Downgrading referred to a decrease in ISUP grade, suggesting the biopsy overestimated the cancer’s severity. Concordance was defined as identical ISUP grades in both biopsy and prostatectomy specimens.

Statistical analyses were performed using GraphPad Prism version 9. The primary outcomes—concordance, upgrading, and downgrading rates between biopsy and radical prostatectomy specimens—were compared between the TRUS-Bx and ComBx groups. Categorical variables were analyzed using the chi-squared test, while continuous variables were assessed using the independent *t*-test. A two-sided *p*-value of less than 0.05 was considered indicative of statistical significance. Concordance rates were analyzed both for overall ISUP grading and for clinically significant disease, providing a comprehensive evaluation of each biopsy method’s accuracy in predicting final pathology.

Approval from the Medical University of Białystok bioethics committee was obtained in accordance with resolution number APK.002.439.2022 on 15 December 2022.

## 3. Results

The mean age, PSA level, and prostate volume in the TRUS-Bx group were 65.1 years, 8.2 ng/mL, and 57.6 cm^3^, respectively. In the ComBx group, the corresponding values were 64.9 years, 7.5 ng/mL, and 60.5 cm^3^. Palpable abnormalities on digital rectal examination were observed in 22 TRUS-Bx cases and 16 ComBx cases. No statistically significant differences were found between the groups in these parameters.

In the TRUS-Bx group, PCa was diagnosed in 113 of 250 patients (45%), while in the ComBx group, 152 of 250 patients (61%) were diagnosed with PCa (*p* < 0.001). Within these subsets, 89 patients from the TRUS-Bx group and 68 from the ComBx group proceeded to RP at our institution. To assess the diagnostic accuracy of each biopsy technique, ISUP grades assigned from biopsy specimens were compared with those determined from the corresponding radical prostatectomy specimens, with a focus on identifying discrepancies in PCa grading.

Concordance between ISUP grade assigned at biopsy and grade determined from radical prostatectomy specimens was observed in 44 of 89 patients (49%) in the TRUS-Bx group and in 43 of 68 patients (63%) in the ComBx group, with a statistically significant difference between the two methods (*p* = 0.042).

Upgrading from the biopsy to prostatectomy specimen was observed in 31 of 89 patients (35%) in the TRUS-Bx group and in 11 of 68 patients (16%) in the ComBx group, also demonstrating a statistically significant difference (*p* = 0.004).

**Figure 2:**
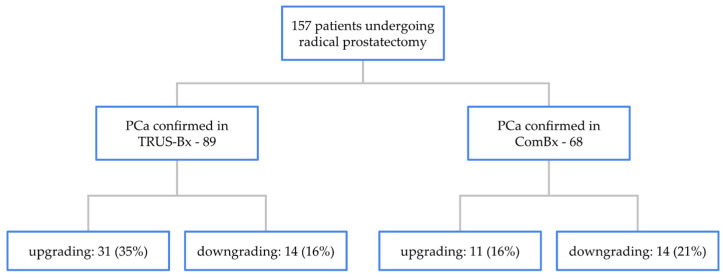
Distribution of upgrading and downgrading rates between biopsy and radical prostatectomy according to biopsy method.

Downgrading occurred in 14 of 89 patients (16%) in the TRUS-Bx group and in 14 of 68 patients (21%) in the ComBx group, with no statistically significant difference between groups (*p* = 0.215). The rates of upgrading and downgrading are illustrated in Figure 2.

Reported complications following biopsy differed between the two groups. The TRUS-Bx group exhibited a higher incidence of infectious complications, including urinary tract infections and cases of sepsis, likely due to the transrectal route allowing potential contamination from rectal flora. In contrast, the ComBx group, which employed a transperineal approach, demonstrated a significantly lower rate of infectious events but a higher frequency of perineal discomfort. Both biopsy techniques were associated with minor complications such as hematuria and hematospermia, which occurred with comparable frequency across groups. Although these adverse events are clinically relevant, specific complication rates were not quantitatively assessed in this study.

## 4. Discussion

Accurate PCa grading at the time of biopsy is critically important for selecting appropriate treatment strategies, thereby balancing therapeutic effectiveness against potential side effects and complications associated with overtreatment or undertreatment. High concordance minimizes both undertreatment, which may lead to disease progression, and overtreatment, which can expose patients to unnecessary risks. Therefore, refining biopsy techniques is essential for optimizing patient care and improving long-term survival outcomes [10,11].

Our study found that ComBx, which incorporates MRI-targeted sampling alongside systematic cores, significantly outperforms the traditional TRUS-Bx in terms of histopathological concordance with RP specimens.

The primary clinical implication of these findings lies in the markedly reduced upgrading rate observed with the ComBx approach. Specifically, upgrading was documented in only 16% of ComBx patients compared to 35% in the TRUS-Bx group. This finding aligns with previous studies, such as those by Sussman et al., who reported a lower upgrading rate in patients undergoing MRI-targeted biopsy compared to systematic biopsy alone (10% vs. 22%) [12]. Ahdoot et al. reported the lowest upgrading rates with ComBx (3.5%), compared to TBx alone (8.7%) and systematic biopsy alone (16.8%) [7]. Moreover, Diamand et al. demonstrated that ComBx significantly reduced upgrading rates compared to systematic biopsy alone (24% vs. 49%), in the regard that TBx alone showed a 35% upgrading rate, highlighting the critical role of combining TBx and SBx to accurately assess PCa grade [8]. Additionally, Burk et al. emphasized the critical value of systematic sampling in improving grading accuracy, as systematic biopsies frequently identified upgrading cases near targeted biopsy sites. Notably, 62.5% of these proximal misses involved ISUP ≥ 3 cancers, highlighting the potential risk of underestimating tumor aggressiveness when relying solely on TBx [9].

Notably, in our study, ComBx showed a higher histopathological concordance between biopsy results and radical prostatectomy compared to TRUS-Bx (63% vs. 49%, *p* = 0.042). This finding is consistent with studies by Ahdoot et al., who reported the highest histopathological concordance for ComBx among various biopsy techniques [7], and Zattoni et al., who observed improved concordance and lower upgrading rates with transperineal targeted biopsies compared to transrectal targeted approaches [13]. Similarly, Kılıç et al. found that MRI-targeted biopsy techniques—including cognitive fusion, software fusion, and in-bore methods—achieved higher concordance and lower upgrading rates compared to systematic biopsy alone [14]. These findings underscore the critical role of TBx, particularly when combined with systematic sampling, in enhancing the accuracy of PCa grading. However, no single targeted approach has demonstrated clear superiority over the others [5]. Several studies further support the diagnostic superiority of ComBx over TBx alone or systematic biopsy alone, highlighting that patients with an ISUP 1 score on biopsy are particularly prone to upgrading upon final pathology [15,16,17,18].

Interestingly, the downgrading rates were comparable between biopsy methods in our study, with no statistically significant differences. This indicates that although ComBx substantially mitigates the risk of underestimating tumor aggressiveness, its capacity to reduce the overestimation of tumor grade appears less pronounced. This indicates that while ComBx improves grading accuracy, it does not significantly affect downgrading likelihood. This finding aligns with Sussman et al., who also reported no significant difference in downgrading rates between targeted and systematic biopsy approaches [12]. Conversely, a meta-analysis by Weinstein et al. demonstrated a significantly higher downgrading rate following ComBx compared to systematic biopsy alone [19]. Further evidence is provided by Rapisarda et al., who found that the combination of fusion-targeted and systematic biopsies resulted in a 73.6% concordance with final Gleason scores and a 67.3% match in lesion location compared to radical prostatectomy specimens, highlighting the diagnostic reliability of the ComBx technique [20].

Several factors may account for the superior concordance and reduced upgrading observed with the ComBx approach. Foremost is the enhanced targeting precision offered by MRI fusion technologies, enabling biopsies to focus accurately on suspicious lesions identified through advanced MRI sequences, notably multiparametric MRI utilizing T2-weighted, diffusion-weighted, and dynamic contrast-enhanced imaging. Additionally, systematic cores collected during the transperineal biopsy further increase the probability of detecting clinically significant prostate cancer, especially in areas adjacent to targeted lesions, reducing sampling errors associated with lesion heterogeneity.

It is important to emphasize that transperineal biopsy offers substantial advantages in terms of patient safety, particularly by significantly reducing the risk of infectious complications compared to the transrectal approach. Although the perineal route has historically been associated with increased discomfort and a greater need for effective pain management, these challenges can be effectively addressed using established local anesthesia protocols. In our experience, transperineal biopsy is a safe and well-tolerated procedure that can be routinely performed under local anesthesia in an outpatient setting. Although exact complication rates were not calculated in this study, we consider the technique to have a low overall impact on patient well-being, making it a practical and safe option—especially for individuals at elevated risk of infection. This view is further supported by recent findings from the ProBE-PC randomized trial, which reported no significant difference in 30-day infectious or noninfectious complication rates between transperineal and transrectal approaches (2.7% vs. 2.6%, respectively), confirming the safety and feasibility of the transperineal technique even under local anesthesia in office-based settings [21]. Accordingly, the overall safety profile of the ComBx transperineal approach remains favorable. It should also be acknowledged that the implementation of MRI–ultrasound fusion biopsy requires dedicated equipment and operator training and entails a learning curve that may initially affect workflow efficiency and diagnostic consistency.

Despite the clear advantages observed, this study has several limitations that warrant consideration. First, the inherent variability in MRI interpretation and lesion classification may introduce observer bias, potentially influencing biopsy outcomes. While the use of standardized PI-RADS criteria helps reduce inter-reader variability, it does not eliminate it entirely. Additionally, certain benign conditions can mimic prostate cancer both clinically and radiologically, occasionally yielding PI-RADS 4 or 5 scores despite their non-malignant nature; granulomatous prostatitis is a notable example, often presenting with elevated PSA and imaging features highly suggestive of malignancy [22]. This overlap presents a diagnostic challenge, particularly in histopathological evaluation, underscoring the importance of accurate tissue diagnosis to avoid misclassification and overtreatment.

Secondly, the study’s single-center, retrospective design may introduce selection bias, potentially limiting generalizability. Moreover, factors such as lesion location, prostate volume, and individual clinician expertise could influence biopsy accuracy, warranting further investigation in larger, multicenter cohorts. Another limitation is that patients were not stratified based on biopsy history; biopsy-naïve individuals and those with prior negative biopsies were analyzed as a single cohort, which may have affected detection rates, as highlighted in previous studies comparing these subgroups. Additionally, while targeted biopsies were obtained directly from MRI-identified lesions, systematic (mapping) biopsies were taken from other regions of the prostate, intentionally excluding the targeted areas. However, because MRI findings were available prior to performing the systematic biopsy, operator awareness of lesion location may have introduced cognitive bias, potentially influencing sampling patterns and limiting the independence of systematic cores.

Future research should expand upon these findings by exploring longer-term patient outcomes, including survival rates, quality of life, and functional outcomes post-treatment based on biopsy-driven therapeutic decisions. Cost-effectiveness analyses comparing the economic and clinical impact of widespread implementation of MRI fusion biopsy techniques are also necessary. Additionally, emerging technologies, such as artificial intelligence and advanced imaging software, represent promising tools for further enhancing biopsy precision and standardizing MRI interpretation.

In summary, our findings robustly support the superiority of the ComBx approach over traditional TRUS-Bx regarding histopathological accuracy and reduced upgrading rates. Ongoing advancements in imaging and biopsy technology promise further refinements, ultimately optimizing patient management and therapeutic outcomes.

## 5. Conclusions

This study demonstrated that transperineal combined fusion biopsy (ComBx), integrating MRI-targeted sampling with systematic biopsies, significantly improves histopathological concordance between biopsy and radical prostatectomy specimens compared to conventional TRUS-Bx. Patients undergoing ComBx exhibited lower rates of histological upgrading, underscoring the method’s enhanced precision in accurately assessing prostate cancer aggressiveness. Although downgrading rates did not differ significantly between biopsy techniques, the superior accuracy of ComBx in grading prostate cancer can significantly impact clinical decision-making, thereby minimizing the risks associated with both overtreatment and undertreatment. Considering these findings, ComBx should be strongly recommended as a standard practice in prostate cancer diagnostic protocols, particularly for patients with clinically suspected significant prostate cancer lesions. Future research efforts should focus on further refining biopsy methodologies, evaluating long-term patient outcomes, and assessing the cost-effectiveness of widespread adoption of MRI-guided fusion biopsy approaches in routine clinical practice.

## Data Availability

Dataset available on request from the authors.
